# Cochlear Implantation in US Military Veterans: A Single Institution Study

**DOI:** 10.1002/oto2.53

**Published:** 2023-05-12

**Authors:** Douglas J. Totten, Abdul Saltagi, Karen Libich, David B. Pisoni, Rick F. Nelson

**Affiliations:** ^1^ Department of Otolaryngology–Head and Neck Surgery Indiana University School of Medicine Indianapolis Indiana USA; ^2^ College of Medicine, Indiana University School of Medicine Indianapolis Indiana USA; ^3^ Department of Audiology Roudebush Veterans' Administration Medical Center Indianapolis Indiana USA; ^4^ Department of Psychological and Brain Sciences Indiana University Bloomington Indiana USA; ^5^ Department of Neurological Surgery Indiana University School of Medicine Indianapolis Indiana USA

**Keywords:** cochlea, cochlear implant, cochlear implantation, hearing loss, inner ear, noise exposure, sensorineural hearing loss

## Abstract

**Objective:**

Military veterans have high rates of noise‐induced hearing loss (NIHL) which is associated with more significant spiral ganglion neuronal loss. This study explores the relationship between NIHL and cochlear implant (CI) outcomes in veterans.

**Study Design:**

Retrospective case series of veterans who underwent CI between 2019 and 2021.

**Setting:**

Veterans Health Administration hospital.

**Methods:**

AzBio Sentence Test, Consonant‐Nucleus‐Consonant (CNC) scores, and Speech, Spatial, and Qualities of Hearing Scale (SSQ) were measured pre‐ and postoperatively. Linear regression assessed relationships between outcomes and noise exposure history, etiology of hearing loss, duration of hearing loss, and Self‐Administered Gerocognitive Exam (SAGE) scores.

**Results:**

Fifty‐two male veterans were implanted at an average (standard deviation) age of 75.0 (9.2) years without major complications. The average duration of hearing loss was 36.0 (18.4) years. The average time of hearing aid use was 21.2 (15.4) years. Noise exposure was reported in 51.3% of patients. Objectively, AzBio and CNC scores 6 months postoperatively showed significant improvement of 48% and 39%, respectively. Subjectively, average 6‐month SSQ scores showed significant improvement by 34 points (*p* < .0001). Younger age, SAGE score ≥17, and shorter duration of amplification were associated with higher postoperative AzBio scores. Greater improvement in AzBio and CNC scores was associated with lower preoperative scores. Noise exposure was not associated with any difference in CI performance.

**Conclusion:**

Despite high levels of noise exposure and advanced age, veterans derive substantial benefits from cochlear implantation. SAGE score ≥17 may be predictive of overall CI outcomes. Noise exposure does not impact CI outcomes.

**Level of Evidence:**

Level 4.

Cochlear implants (CIs) provide substantial benefits to tens of thousands of Americans with hearing loss who have undergone implantation since the first single‐channel implantation in 1961 and the initial Food and Drug Administration approval of CIs in the 1980s.^1^ US military veterans, who receive care through the unique Veterans Health Administration (VHA) model, are 30% more likely to have severe hearing impairment than adults after adjusting for age and current occupation.^2^ While the Veterans Choice Program and new CI programs at VHA hospitals have increased veteran access to CIs, barriers to care continue to persist in veteran and civilian populations alike, as fewer than 10% of adults in the United States who meet CI criteria have received this treatment.^3^, ^4^, ^5^, ^6^ The unique model through which the VHA provides care, the high propensity for these patients to have severe hearing loss, possibly due to a history of noise exposure, and the inherent importance of providing high‐quality care to patients who serve and/or served in the US Armed Forces, provide ample reason to study veteran CI recipients. Furthermore, the high proportion of patients in this population with a history of noise exposure makes this an ideal population to assess the effect of noise exposure on CI outcomes. As noise exposure has been associated with accelerated spiral ganglion nerve (SGN) cell degeneration, it would be plausible to hypothesize that patients with a history of noise exposure experience worse outcomes compared to patients without this exposure.^7^, ^8^ Military veterans are often of advanced age which has previously been associated with decreased, although still positive, CI performance.^9^, ^10^, ^11^, ^12^ Age‐associated cognitive decline has also been associated with worse CI outcomes on some metrics, particularly in noisy environments.^13^, ^14^ Multiple methods of rapid assessment of cognitive status have been developed including the Self‐Administered Gerocognitive Exam (SAGE).^15^ The purpose of this study was to assess patient outcomes at a recently established CI program in a moderately sized metropolitan area, identify patient characteristics that may be predictive of improved CI outcomes in this population, and assess the effect of noise exposure on CI outcomes.

## Methods

After approval from the Richard L. Roudebush VA Medical Center Institutional Review Board (IRB# 13588), records for patients receiving cochlear implantation at an urban VHA facility in a moderately sized metropolitan area from initiation of the program in May 2019 to May 2022 were retrospectively reviewed. Patient age at implantation, educational attainment, gender, laterality, and history of the prior implant were recorded. Additionally, etiology of hearing loss, duration of hearing loss, preoperative testing scores, type of implant and processor, and SAGE scores were recorded along with postoperative AzBio Sentence Test and Consonant‐Nucleus‐Consonant (CNC) scores at 2, 3, 6, and 12 months postoperatively. The SAGE test is “a brief self‐administered cognitive screening instrument to identify Mild Cognitive Impairment and early dementia” with a maximum score of 22.^16^ The SAGE test is administered in written format and is independent of hearing status. SAGE scores ≥17 are considered normal. Patients with SAGE scores <17 are referred for neuropsychological evaluation prior to proceeding with CI. Preoperative tests performed at our VHA center include CNC, AzBio, aided audiometric thresholds, SAGE scores, Speech, Spatial, and Qualities of Hearing Scale (SSQ), and vestibular testing. SAGE scores and International Outcome Inventory for Hearing Aids adapted for cochlear implants (IOI‐CI) scores were recorded 1 year postoperatively. Outcomes were also compared to recently published CI outcomes at a nearby academic medical center that involved the same CI surgeon (senior author).^17^ Outcomes for first versus second implants in patients who received bilateral implants were also assessed.

### Statistical Analysis

Statistical analysis was performed using SAS software (SAS Institute). Single‐predictor linear regression was used to assess the impact of “normal” SAGE scores (ie, scores ≥17), tinnitus, and noise exposure on changes in CNC and AzBio scores in implanted ears at 2, 3, 6, and 12 months postoperatively. Linear regression was also used to assess the effect of daily usage (hours per day) on change in CNC and AzBio scores. Multivariable linear regression was subsequently used to analyze the effect of SAGE scores and daily usage on patient CNC and AzBio scores at the time points mentioned above.

## Results

**Figure 1:**
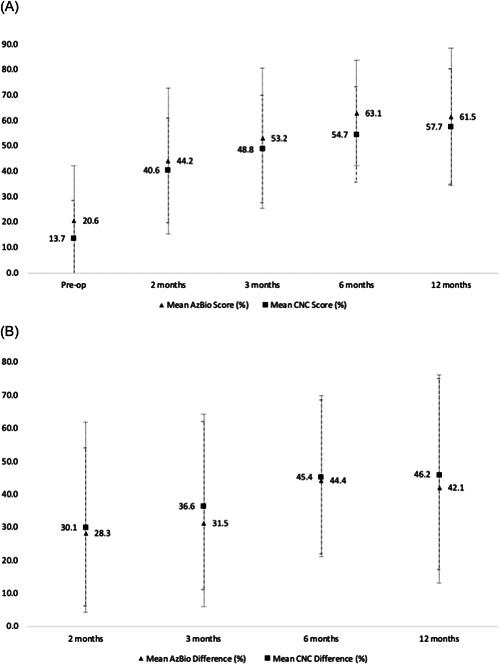
(A) Mean pre‐ and postoperative AzBio and CNC scores at various time points. (B) Mean change in AzBio and CNC scores from preoperative scores at various time points postoperatively. CNC, Consonant‐Nucleus‐Consonant

A total of 52 patients (59 ears) were implanted from the initiation of the CI program in May 2019 to May 2022. The average age at implantation was 75.0 (standard deviation [SD]: 9.2) years, while the median age was 75 years (interquartile range: 70‐80) while 50 (98%) of patients with recorded race were white (Table 1). The average duration of hearing loss was 36.0 (18.4) years while the average duration of hearing aid use was 21.2 (15.4) years. All patients were male. Of 39 patients with hearing loss etiology reported, 20 (51.3%) reported a history of noise exposure. Educational attainment was recorded for 41 patients. Of these, 33 (80.5%) had completed high school but had no further education, while 4 (9.8%) patients did not complete a high school degree. Two patients (4.9%) had received a college diploma without further education while 2 patients (4.9%) had received postgraduate degrees. The average SAGE score was 17.8. A total of 34 patients (73.9%) had a “normal” SAGE score (ie, ≥17). Tinnitus was reported by 30 (58.8%) of patients. Preoperative AzBio score was 20.6% correct word recognition in sentences (SD: 21.7%) and preop CNC score was 13.7% correct word recognition in isolation (14.9%). Postoperative AzBio and CNC scores, and improvement postoperatively, are shown in Figure 1. All ears were implanted with Cochlear^TM^ Nucleus® implants with the CI522 implanted in 15 (25%) ears, the CI622 in 36 (61%) ears, and the CI624 in 8 (14%) ears. The type of electrode was not associated with a significant difference in CNC or AzBio outcomes at any time point. Seven patients received bilateral CIs. The left ear was implanted first in 5 (71%) of the bilaterally implanted patients. Six months after the respective operations, patients recorded a mean (SD) AzBio score of 80.0% (11.9%) in the initially implanted ear and 79.8% (2.1%) in the second implanted ear (*p* = .97). CNC scores 6 months postoperatively were 69.2% (7.0%) for initially implanted ear and 72.5% (7.5%) for subsequently implanted ear (*p* = .52). The average IOI‐CI score was 38.8 (4.0) 1 year postoperatively. Of 8 patients with SAGE score recorded 1 year postoperatively, only 1 patient with a “normal” sage score preoperatively recorded an abnormal SAGE score postoperatively.

**Table 1:** Patient Demographics and Hearing Loss Etiology

	Avg/N	SD/%
Age	75	9
Race: White	51	98.0%
Hearing loss etiology		
Noise exposure	20	51.3%
Unknown	8	20.5%
Presbycusis	9	23.1%
Genetic	2	5.1%
Meniere's	3	7.7%
Viral	1	2.6%
Surgical failure	1	2.6%
Infections	1	2.6%
Duration hearing loss	36.3	18.7
Years aided	21.6	16.0
Education		
Some HS	4	9.8%
HS graduate	33	80.5%
College graduate	4	9.8%

Abbreviations: Avg, average; HS, high school; N, number; SD, standard deviation.

Single‐variable linear regression was used to assess AzBio and CNC score outcomes and improvement postoperatively at 2, 3, 6, and 12 months postoperatively. Factors associated with significantly better AzBio performance postoperatively were: younger age at 2, 3, 6, and 12 months, SAGE score ≥17 at 2, 3, and 6 months, tinnitus at 3 and 12 months, and duration of amplification and duration of hearing loss at 6 months (Table 2). A better CNC score 2 months postoperatively was associated with younger age and shorter duration of amplification. No other factors were significant for greater CNC score at any time point postoperatively. SSQ scores improved from a mean (SD) of 32.8 (17.9) to 63.3 (17.6) 6 months postoperatively (*p* < .0001).

**Table 2:** Significant Predictor Variables at 2‐, 3‐, 6‐, and 12‐Month Postoperative Time Points for Absolute AzBio and CNC Scores

Test	Time point, mo	Significant predictors	Estimate	*p* value
AzBio	2	Younger age	−1.7	<.0001
		SAGE ≥ 17	27.1	.0099
	3	Younger age	−1.2	.0037
		SAGE ≥ 17	28.4	.0031
		Tinnitus	17.4	.0457
	6	Younger age	−1.0	.0013
		Duration amplification	−0.4	.0425
		Duration hearing loss	−0.4	.0072
		SAGE ≥ 17	14.9	.0472
	12	Younger age	−1.6	.0005
		Tinnitus	22.7	.018
CNC	2	Younger age	−0.7	.0337
		Duration amplification	−0.4	.0448

Abbreviations: CNC, Consonant‐Nucleus‐Conconant; SAGE, Self‐Administered Gerocognitive Exam.

Factors associated with a greater improvement in AzBio score were: younger age at 2 and 12 months, shorter duration of amplification at 2 months, duration of hearing loss at 6 months, and worse preoperative AzBio score at all time points (Table 3). A shorter duration of amplification was associated with greater improvement at 2 months. Worse preoperative CNC score was associated with greater improvement in postoperative CNC score compared to preoperative score at all time points while shorter duration of amplification was associated with greater improvement in CNC score 2 months postoperatively. The effect of noise exposure was also assessed using single‐predictor linear regression. Noise exposure was not found to be significantly associated with worse CI performance or any difference in AzBio or CNC score improvement postoperatively (Table 4). Multivariable linear regression was also performed using the above variables without identifying any factors with a significant impact on AzBio or CNC scores or differences. Postoperative AzBio scores in this veteran population were not significantly different (*p* = .79) from the civilian population despite having an older mean age (75 vs 62) and a much higher rate of noise exposure (51.3% vs 15.7%).

**Table 3:** Significant Predictor Variables at 2‐, 3‐, 6‐, and 12‐Months Postoperatively for Change in AzBio and CNC Scores

Test	Time point, mo	Significant predictors	Estimate	*p* value
AzBio	2	Younger age	−1.5	.0027
		Duration amplification	−0.9	.0138
		Preop AzBio	−0.9	.0003
	3	Preop AzBio	−0.8	<.0001
	6	Duration hearing loss	−0.4	.0478
		Preop AzBio	−0.7	<.0001
	12	Younger age	−1.4	.0437
		Preop AzBio	−0.7	.0042
CNC	2	Duration amplification	−0.6	.0265
		Preop CNC	−0.7	.0041
	3	Preop CNC	−0.8	.0009
	6	Preop CNC	−0.9	.0001
	12	Preop CNC	−1.4	<.0001

Abbreviations: CNC, Consonant‐Nucleus‐Conconant; Preop, preoperative.

**Table 4:** Effect of Noise on Absolute and Change in AzBio and Consonant‐Nucleus‐Conconant (CNC) Scores

Test	Noise and AzBio score			Noise and change in AzBio score		
	Time point, mo	Estimate	*p* value	Time point, mo	Estimate	*p* value
AZBio	2	−27.6	.23	2	−17.6	.47
	3	−22.6	.32	3	−19.4	.44
	6	−13.4	.43	6	−7.9	.73
	12	N/A	N/A	12	N/A	N/A
CNC	2	−8.7	.60	2	−1.0	.96
	3	−18.6	.31	3	−13.1	.51
	6	−35.3	.06	6	−25.5	.23
	12	N/A	N/A	12	N/A	N/A

Abbreviation: N/A, not applicable.

## Discussion

Cochlear implantation is highly effective in treating moderate‐to‐profound hearing loss in a sample of elderly male Veterans. CI enables patients to detect and understand sound and speech, therefore providing hearing‐impaired patients the ability to hear, communicate, and interact with the world around them. This study provides unique insight into hearing rehabilitation via cochlear implantation in a unique understudied population with a high proportion of reported noise‐induced hearing loss (NIHL) who receive care through the VHA.

The VHA provides high‐quality health care to millions of veterans across the United States. A previous study demonstrated that veterans lived a median of 80 miles from the nearest VHA facility offering cochlear implantation and associated care.^3^ However, in recent years, multiple facilities, including the one analyzed in this study, have initiated a CI program. The availability of cochlear implantation locally has provided many veterans with additional opportunities to receive high‐quality otologic and audiologic care through the VHA. Additionally, the VHA has provided remote programming services in addition to travel and lodging assistance to patients who would otherwise be required to travel long distances to receive care.^5^ This study demonstrates that outcomes at a recently established CI program at a VHA facility are comparable to those at a local academic medical center despite patients being older and having a greater percentage of patients with a history of noise exposure. Although advanced age and prolonged duration of hearing loss may reduce overall CI outcomes, this study documents that patients of advanced age and extended duration of hearing loss may derive significant benefit from implantation, particularly as our patients displayed, on average, a near‐doubling of overall SSQ score by 6 months after implantation, suggesting substantial improvement in hearing outcomes and quality of life.^18^, ^19^

History of NIHL was also not predictive of CI outcomes among veterans. Although noise exposure has been associated with accelerated SGN cell death, likely due to loss of trophic support after hair cell death, patient outcomes are equivalent to those without a noise exposure history. This is consistent with prior research suggesting that SGN counts did not correlate with CI outcomes when the SGN counts were between 2800 to 25,000. However, counts <2800 were associated with poor CI outcomes.^20^ As noise exposure does not adversely affect outcomes in veterans—who are at higher risk of experiencing severe acoustic trauma than almost any other population—NIHL appears unlikely to reduce SGN counts below the level necessary to worsen CI outcomes in any population. Thus, a history of noise exposure alone should not impact decision‐making regarding candidacy for cochlear implantation.

Factors that were predictive of better and/or more improved AzBio or CNC scores at various postoperative time points included “normal” SAGE cognition scores, younger age, and shorter duration of amplification. These findings are consistent with prior studies, which have previously demonstrated these relationships.^21^ In our practice, all CI candidates with SAGE scores less than 17 are referred for neuropsychological evaluation prior to proceeding with implantation. While most patients were able to proceed with implantation with familial support, 2 patients with SAGE scores of 7 or less were not offered CI based on neuropsychological evaluation. Additionally, 2 patients with SAGE scores less than 7 elected not to undergo neuropsychological testing and withdrew from CI candidacy. Having a worse preoperative AzBio or CNC score was also predictive of greater improvement in postoperative word recognition scores. This suggests that a worse preoperative test is not indicative of poor prognosis, but rather of a greater possibility for improvement. For bilateral implantees, no relationship was observed between the order of implantation and CI outcomes.

Limitations to this study include limited duration of follow‐up due in part to the COVID‐19 pandemic, the recent establishment of the CI program resulting in the limited number of patients, and 1 location of a relatively homogenous population making generalizations to the overall population challenging. However, the majority of hearing improvement is seen in the first 3 to 6 months postoperatively.^22^, ^23^ Additionally, this study demonstrates that VHA CI outcomes can be equivalent to nearby academic health centers, younger age, normal cognition, and shorter duration of amplification may positively impact postoperative outcomes, and that a history of noise exposure does not adversely impact CI outcomes.

## Conclusion

NIHL does not adversely impact CI outcomes in military veterans. As veterans are at risk of exposure to levels of acoustic trauma greater than almost any other population, NIHL is unlikely to adversely impact CI outcomes in any population. Consistent with previous literature, younger age, improved cognition, and shorter duration of hearing loss may improve CI outcomes in military veterans.

## Author Contributions

**Douglas J. Totten**, study development and design, data collection, data analysis, manuscript composition; **Abdul Saltagi**, data collection, manuscript composition; **Karen Libich**, study development and design, data collection, manuscript composition; **David B. Pisoni**, study development and design, manuscript composition; **Rick F. Nelson**, study development and design, manuscript composition.

## Disclosures

### Competing interests

None.

### Funding source

None.
